# Changes in intestinal microbiota in HIV-1-infected subjects following cART initiation: influence of CD4+ T cell count

**DOI:** 10.1038/s41426-018-0117-y

**Published:** 2018-06-22

**Authors:** Yongjia Ji, Fengdi Zhang, Renfang Zhang, Yinzhong Shen, Li Liu, Jiangrong Wang, Junyang Yang, Qi Tang, Jingna Xun, Tangkai Qi, Zhenyan Wang, Wei Song, Yang Tang, Jun Chen, Hongzhou Lu

**Affiliations:** 1 Department of Infectious Disease, Shanghai Public Health Clinical Center, Fudan University, Shanghai, 201508 China; 2 Department of Infectious Disease, Huashan Hospital Affiliated to Fudan University, Shanghai, 200040 China; 3 Department of Internal Medicine, Shanghai Medical College, Fudan University, Shanghai, 200032 China

## Abstract

The roles of immunodeficiency and combined antiretroviral therapy (cART) in shaping the gut microbiota in HIV-1-infected subjects (HISs) have not been described thoroughly by time-series investigations. In this study, 36 antiretroviral-naïve HISs were enrolled to prospectively assess alterations in the fecal microbiota and plasma markers of microbial translocation and inflammation with cART. At baseline, the species α-diversity of the fecal microbiota was significantly lower in HISs with a CD4^+^ T cell count <300/mm^3^ than in HISs with a CD4^+^ T cell count >300/mm^3^ (Shannon index: Median 2.557 vs. 2.981, *P* = 0.006; Simpson index: Median 0.168 vs. 0.096, *P* = 0.004). Additionally, the baseline α-diversity indices correlated with CD4^+^ T cell counts (Shannon index: *r* = 0.474, *P* = 0.004; Simpson index: *r* = −0.467, *P* = 0.004) and the specific plasma biomarkers for microbial translocation and inflammation. After cART introduction, the species α-diversity of fecal microbiota in HISs with CD4^+^ T cell counts <300/mm^3^ was significantly restored (Shannon index: Median 2.557 vs. 2.791, *P* = 0.007; Simpson index: Median 0.168 vs. 0.112, *P* = 0.004), while the variances were insignificant among HISs with CD4+ T cell counts >300/mm^3^ (Shannon index: Median 2.981 vs. 2.934, *P* = 0.179; Simpson index: Median 0.096 vs. 0.119, *P* = 0.082). Meanwhile, with cART introduction, alterations in the gut microbial composition were more significant in the subgroup with CD4^+^ T cell counts >300/mm^3^, corresponding to increases in the specific plasma inflammatory markers. These findings implicated the interactive roles of immunodeficiency and cART for affecting gut microbiota in HIV-1-infected individuals, providing new insights into intestinal microbiome dysbiosis related to HIV-1 infection.

## Introduction

There are an estimated 10–100 trillion microorganisms inhabiting the human gastrointestinal tract^1^. The symbiotic relationship that is established between the intestinal microbiota and the host plays a crucial role in maintaining host health^2^. Human immunodeficiency virus type 1 (HIV-1) preferentially infects and destroys CD4^**+**^ T lymphocytes^3^. Even during early HIV-1 infection, the T helper cells residing in gut-associated lymphoid tissue are depleted, which induces disintegration and dysfunction of the intestinal mucosal barrier^4^. Consequently, with the loss of protection by the intestinal mucosal barrier, microbial products (such as lipopolysaccharides) can intrude into the lamina propria of the gut and the systemic circulation in a process known as microbial translocation^5,6^. In addition, the composition of the intestinal microbiota shifts in HIV-1-infected individuals compared with that in non-HIV-infected individuals^7^, which is an indicator of host immune status^8^.

Combined antiretroviral therapy (cART) effectively inhibits HIV-1 replication in the host. However, even with sustained viral suppression, a chronic immune activation and hyper-inflammatory state manifests in HIV-1-infected individuals^9^, and this state may be associated with the increased incidence of specific diseases such as cancer, metabolic disorders and cardiovascular disease^10,11^. Given that the gut microbiota is closely linked to the host’s immune profile, the microbial dysbiosis in HIV-1-infected individuals likely significantly contributes to the excessive risk of inflammation-related diseases^12^. Considering that the findings from previous cross-sectional studies may be biased by confounding factors that impact the gut microbiome, such as dietary patterns and male homosexual behaviors^13,14^, the roles of the host’s immune status, and cART on the intestinal microbiota in HIV-1-infected subjects have not been clearly determined^15–18^. Hence, we present this study with the aim of prospectively observing the impact of short-term cART on the intestinal microbiota of HIV-1-infected subjects with varying immune statuses.

## Materials and methods

### Study settings and design

This prospective cohort study was performed at the Shanghai Public Clinical Health Center (SPHCC) affiliated with Fudan University from December 2015 to June 2017. Thirty-six HIV-1-infected subjects (HISs) were consecutively enrolled. At the time of enrollment, all HISs were older than 18 years and had not initiated cART. The exclusion criteria included inflammatory bowel disease, infectious gastroenteritis, antibiotic/probiotic administration within 4 weeks prior to sample collection and HIV-related complications.

At the time of recruitment, both fecal and blood samples were collected from the HISs. Thereafter, all HISs were given cART (tenofovir, lamivudine, and efavirenz) according to the current recommendations in China and underwent regular follow-up. Approximately 12 months later, the HISs were recalled to collect fecal and blood samples. The study was approved by the SPHCC Institutional Review Board, and informed consent was obtained from all participants.

### Laboratory assays for T cell populations, HIV viral, load and plasma biomarkers of microbial translocation and inflammation

Immediately after sample collection, plasma and peripheral blood mononuclear cells (PBMCs) were separated from whole blood and stored at −80 °C for batch assays. T cell population assessments and plasma HIV-1 RNA load assays were performed by using flow cytometry and Cobas Amplicor (Roche, USA) at the clinical diagnostic laboratory of the SPHCC. Customized kits (MILLIPLEX® MAP; Merck Millipore, Germany) were used to quantify 38 cytokines in the plasma samples (Supplementary Table 1). In addition, enzyme-linked immunosorbent assays (ELISAs) were performed to quantify plasma markers for microbial translocation, including endotoxin core immunoglobulin M (EndoCAb IgM, Hycult Biotech, The Netherlands), lipopolysaccharide binding protein (LBP, Hycult Biotech, The Netherlands), soluble CD14 (sCD14, R&D Systems, USA), soluble CD163 (sCD163, R&D Systems, USA), and intestinal fatty acid-binding protein (I-FABP, R&D Systems, USA). All assays were performed following the manufacturer’s instructions. Measured values below the limit of detection were considered zero. For values beyond the detection range, the value of the upper limit was assigned.

### DNA extraction, amplification, and 16S rRNA gene sequencing

Fecal samples were stored at -80 degrees C after collection until DNA extraction. The samples were processed, and DNA was extracted using an EZNA® Stool DNA Kit (Omega Bio-Tek, USA) according to the manufacturer’s instructions. 16S rRNA gene libraries were established according to the 16S Metagenomic Sequencing Library Preparation Guide (Illumina, USA). The targeted V3-V4 region of the 16S rRNA gene from extracted DNA was PCR-amplified with the universal primers 341F (5′-AGAGTTTGATCMTGGCTCAG-3′) and 805R (5′-GACTGGAGTTCCTTGGCACCCGAGAATTCCA-3′). The PCR products were purified by AMPure XP® beads (Beckman Coulter, UK), and each PCR amplicon was quantified using Qubit 2.0 (Life Technologies, USA) and pooled at equal concentrations for sequencing. 16S rRNA gene sequencing was performed on an Illumina MiSeq platform (Illumina, USA) at Sangon Biotech in Shanghai, China.

### Sequence analyses

All reads were demultiplexed, preprocessed, and subsequently analyzed with the Quantitative Insights into Microbial Ecology (QIIME) software package ver. 1.8.0. Sequence numbers were randomly subsampled to 10,000 per sample to ensure an even amount of information for analysis. Operational taxonomic units (OTUs) were generated using USEARCH software version 7.0 (http://www.drive5.com/uparse/) at a similarity threshold of 97%. The taxonomic classification was constructed using the Bayesian classifier with Silva database 128 (http://www.arb-silva.de) as a reference.

### Statistical and bioinformatic analysis

For cross-sectional analyses of characteristics and diversity indices between groups, differences were estimated by Student’s *t*-test, the Mann–Whitney *U* test or the chi-square test, as appropriate. For comparisons between baseline and follow-up samples, a paired *t*-test or Wilcoxon signed-rank test was performed depending on the distribution of the variable. Each correlation analysis was assessed using nonparametric Spearman’s rank tests and corrected by the Benjamini and Hochberg false discovery rate (BH FDR) test. Multivariate linear regression models were constructed to examine the factors associated with α-diversity indices. Two-tailed significance was set at *P* < 0.05.

The species richness and α-diversity of the fecal microbiome were indicated by the number of observed species (SOB) and Shannon and Simpson indices. β-diversity was assessed by principal coordinate analysis (PCoA). Weighted UniFrac distances were utilized to estimate the sample distributions in each group, and an Adonis significance analysis was performed for each pairwise comparison.

To identify the differentially abundant bacterial taxa between groups, linear discriminant analyses (LDA) coupled with effect size measurements (LefSe) were performed. The primary bacterial taxa for differentiating between groups were identified as those taxa with relative abundances >0.1% (in any group) showing an LDA score > 4, which were further tested using nonparametric paired Wilcoxon signed-rank tests (*P* < 0.05).

Statistical analyses were performed using GraphPad Prism version 7.0 (GraphPad Software, USA), SPSS version 22 (IBM, USA), and the R programming environment (http://www.r-project.org/).

## Results

### Clinical and demographic characteristics of the participants

The demographic and clinical characteristics of the 36 HISs in this cohort are described in Table 1. At baseline in this study, all HISs were cART-naïve with detectable plasma viral loads (median 4.68lg10 copies/ml, IQR 4.18–5.12lg10 copies/ml), and most patients exhibited moderate immunodeficiency (median CD4^**+**^ T cell count: 331 cells/mm^3^, IQR: 247–429 cells/mm^3^). After their enrollment, the median follow-up duration was 15 months (IQR: 14–16 months). At the last visit, 32 patients (88.9%) had undetectable viral loads, and the remaining 4 patients had extremely low levels (median: 41 copies/ml, range: 20–96 copies/ml). The median recovery of CD4^**+**^ T cells from baseline was 138 cells/mm^3^ (IQR: 87–248 cells/mm^3^). When all enrolled subjects were divided by baseline CD4^**+**^ T cell count (300/mm^3^), there were no significant differences in age (*P* = 0.791), gender (*P* = 0.715), sexual preference (*P* = 0.760), baseline HIV viral load (*P* = 0.234), and cART duration (*P* = 0.968) between the two subgroups (Table 1).

**Table 1:** Demographics and clinical characteristics of participants

	Overall *N* = 36	CD4^+^ T cells <300/mm^3^ *N* = 14	CD4^+^ T cells >300/mm^3^ *N* = 22	*P* value^a^
Age, years, median (IQR)	33 (29–40)	32 (29–38)	33 (29–44)	0.791^b^
Male gender, *N* (%)	34 (94.4%)	13 (92.9%)	21 (95.5%)	0.715^c^
Man who have sex with man, *N* (%)	30 (83.3%)	12 (85.7%)	18 (81.8%)	0.760^c^
Baseline CD4^+^ T cell/mm^3^, median (IQR)	331 (247–429)	227 (150–263)	414 (345–484)	<0.001^b^
Last visit CD4^+^ T cell/mm^3^, median (IQR)	467 (342–619)	370 (272–454)	581 (400–792)	0.001^d^
Baseline CD4^+^/CD8^+^ ratio, %, median (IQR)	39.57 (25.33–39.57)	27.22 (14.48–42.49)	52.87 (32.12–67.93)	0.014^d^
Last visit CD4^+^/CD8^+^ ratio, %, median (IQR)	63.12 (48.64–93.49)	55.30 (39.83–62.98)	89.45 (53.90–100.07)	0.005^d^
Baseline HIV RNA lg10 copies/ml, median (IQR)	4.68 (4.18–5.12)	4.70 (4.25–5.41)	4.66 (3.62–5.08)	0.234^d^
cART duration, months, median (IQR)	15 (14–16)	15 (14–16)	15 (14–17)	0.968^b^

^a^Compared between subjects with CD4^+^ T cells <300/mm^3^ and >300/mm^3^

^b^Analyzed by Mann–Whitney *U* test

^c^Analyzed by chi-square test

^d^Analyzed by *t*-test

### Baseline species α-diversity of fecal microbiota correlated with CD4^+^ T cell counts and plasma biomarkers for microbial translocation and inflammation in HISs

**Fig. 1:**
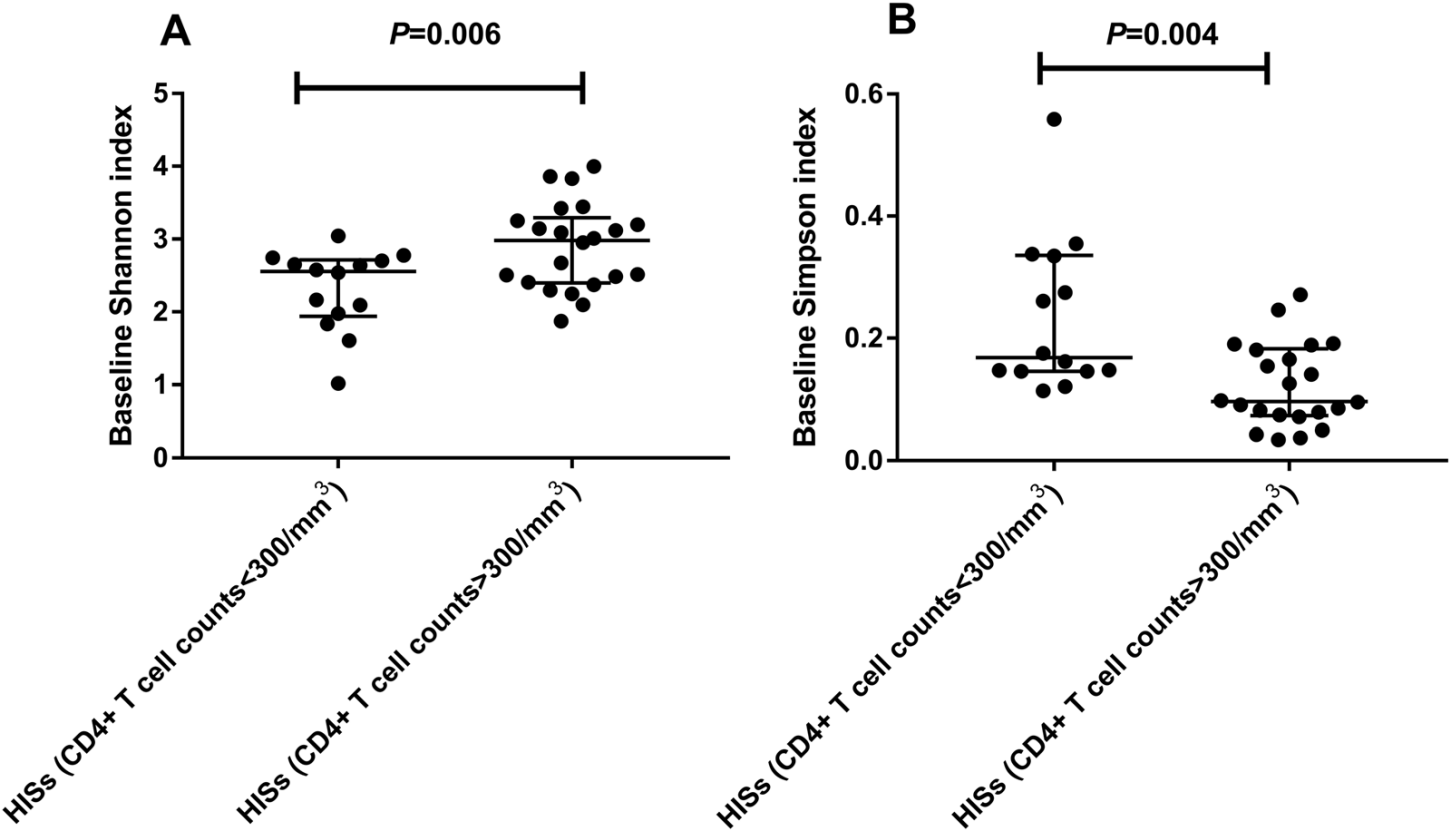
Differences in species α-diversity of fecal microbiota between HISs subgrouped by CD4^**+**^ T cell counts at 300/mm^3^. **a** Shannon index: *t* test; **b** Simpson index: Mann–Whitney test

At baseline, the species α-diversity of fecal microbiota was significantly lower in HISs with a CD4^**+**^ T cell count <300/mm^3^ than in HISs with a CD4^+^ T cell count >300/mm^3^ (Fig. 1), with a decrease in the Shannon index (Median 2.557 vs. 2.981, *P* = 0.006) as well as an increase in the Simpson index (Median 0.168 vs. 0.096, *P* = 0.004). However, the species richness index (SOB) did not significantly differ between the two subgroups at baseline (Median 102 vs. 120, *P* = 0.256).

The bivariate correlation analysis revealed that the Shannon index was positively correlated with the CD4^**+**^ T cell count (*r* = 0.474, *P* = 0.004) and negatively correlated with plasma markers for microbial translocation and inflammation at baseline, including lipopolysaccharide binding protein (LBP; *r* = −0.389, *P* = 0.028), fibroblast growth factor (FGF)-2 (*r* = −0.391, *P* = 0.022), interferon (IFN)-α2 (*r* = −0.418, *P* = 0.014), interleukin (IL)-1β (*r* = −0.342, *P* = 0.048), IL-3 (*r* = −0.345, *P* = 0.046), IL-4 (*r* = −0.382, *P* = 0.026), IL-7 (*r* = −0.379, *P* = 0.027), IL-9 (*r* = −0.368, *P* = 0.032), IL-15 (*r* = −0.402, *P* = 0.019), and macrophage inflammatory protein (MIP)-1β (*r* = −0.390, *P* = 0.023). Conversely, the baseline Simpson index was negatively correlated with the CD4^**+**^ T cell count (*r* = −0.467, *P* = 0.004) and positively correlated with LBP (*r* = 0.425, *P* = 0.015), FGF2 (*r* = 0.357, *P* = 0.038), IFN-α2 (*r* = 0.398, *P* = 0.020), IL-4 (*r* = 0.443, *P* = 0.009), IL-6 (*r* = 0.353, *P* = 0.041), IL-10 (*r* = 0.352, *P* = 0.041), IL-15 (*r* = 0.411, *P* = 0.016), and MIP-1β (*r* = 0.460, *P* = 0.006). After the BH FDR correction with a threshold at *q* < 0.2, the CD4^**+**^ T cell count (*q* = 0.176), LBP (*q* = 0.152), IL-9 (*q* = 0.158), IL-7 (*q* = 0.171), IL-4 (*q* = 0.190), and IL-1β (*q* = 0.192) were significantly correlated with the Shannon index, and the CD4^**+**^ T cell count (*q* = 0.176), IL-4 (*q* = 0.127), MIP-1β (*q* = 0.136), IL-15 (*q* = 0.139), IFN-α2 (*q* = 0.145), and LBP (*q* = 0.169) were significantly correlated with the Simpson index.

During multivariate linear regression analysis, the baseline CD4^**+**^ T cell count (standardized β-coefficient = 0.499, *P* < 0.001), IL-7 (standardized β-coefficient = −0.524, *P* < 0.001), and EndoCab IgM (standardized β-coefficient = −0.273, *P* = 0.038) were identified as factors that independently correlated with the Shannon index. Additionally, the baseline CD4^**+**^ T cell count (standardized β-coefficient = −0.590, *P* < 0.001) and IL-7 (standardized β-coefficient = 0.494, *P* < 0.001) were independently correlated with the Simpson index.

### cART introduction affected gut microbiome diversity and plasma markers for inflammation and microbial translocation in HISs

**Fig. 2:**
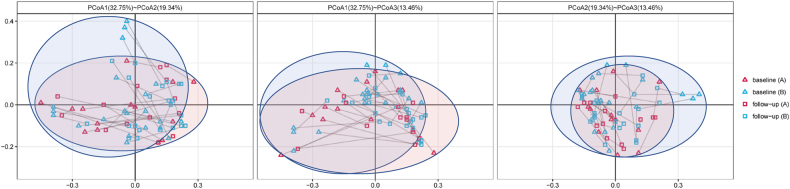
**Characterization of β-diversity of intestinal microbiota in HISs at baseline and follow-up by PCoA (the paired baseline and follow-up samples were connected with lines. a** HISs with baseline CD4^+^ T cell counts <300/mm^3^; **b** HISs with baseline CD4^+^ T cell counts >300/mm^3^

Overall, cART introduction did not impact the species richness (SOB: Median 114 vs. 110 *P* = 0.952) and α-diversity (Shannon index: Median 2.645 vs. 2.890, *P* = 0.589; Simpson index: Median 0.146 vs. 0.121, *P* = 0.560) of the gut microbiota in HISs in this cohort. For β-diversity (Fig. 2), a statistically significant difference was observed in the Adonis analysis comparing the microbial community profiles of the paired baseline and cART-treated samples (Adonis analysis of weighted UniFrac distance metric, *P* = 0.001). Additionally, microbial translocation markers, including sCD163 (median 893.70 vs. 596.70 ng/ml, *P* < 0.001) and I-FABP (median 20.14 vs. 9.20 ng/ml, *P* < 0.001), significantly decreased with alterations in specific inflammatory biomarkers (Supplementary Table 2) after cART.

**Fig. 3:**
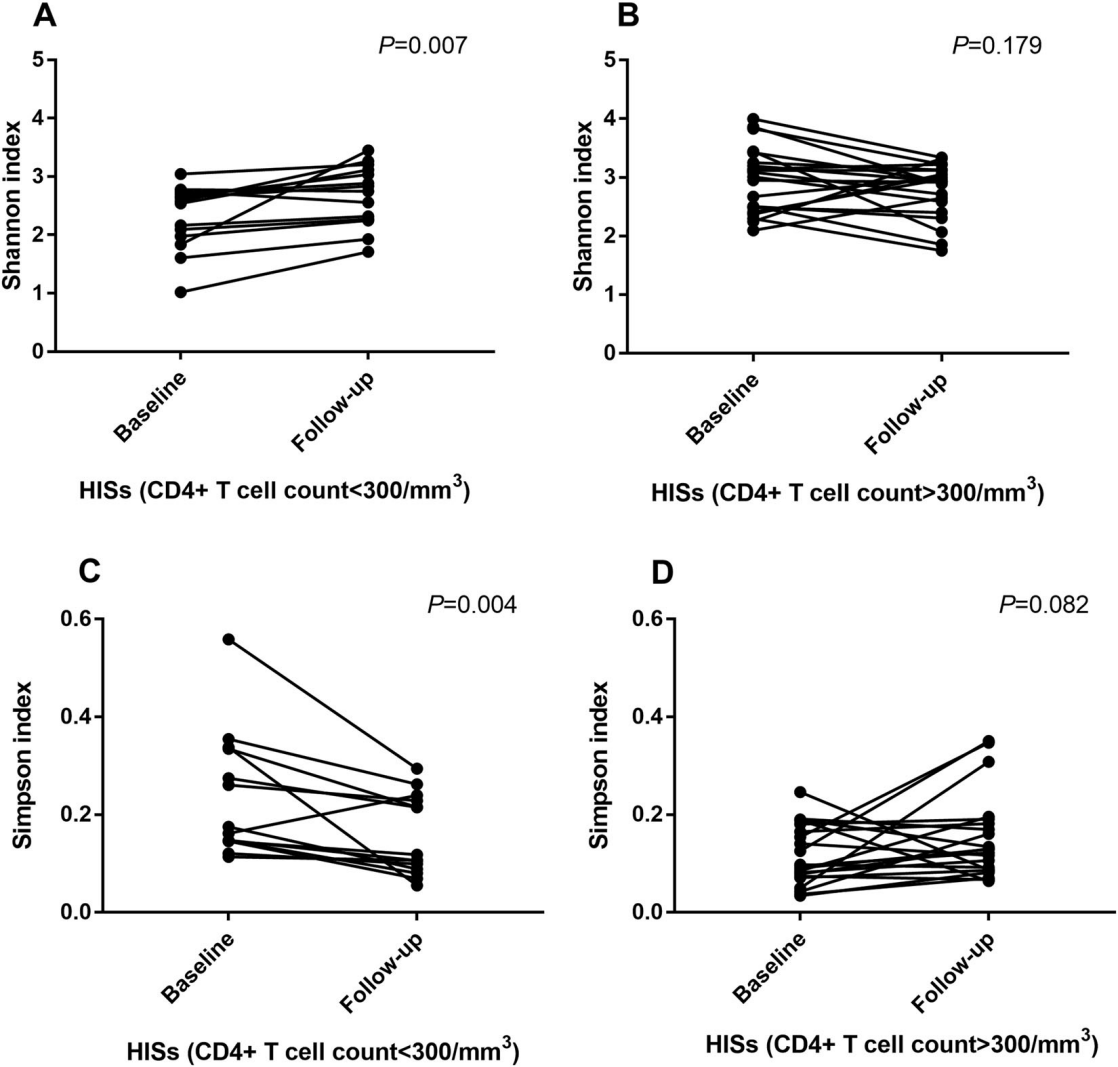
**Paired comparisons (paired*t*** **test) of α-diversity indices in HISs before and after cART treatment. a** Analysis of the Shannon index in HISs with baseline CD4^+^ T cell counts <300/mm^3^; **b** Analysis of the Shannon index in HISs with baseline CD4^+^ T cell counts >300/mm^3^; **c** Analysis of the Simpson index in HISs with baseline CD4^+^ T cell counts <300/mm^3^; **d** Analysis of the Simpson index in HISs with baseline CD4^+^ T cell counts >300/mm^3^)

In the subgroup analysis of HISs with CD4^**+**^ T cell counts <300/mm^3^ at baseline, the species α-diversity of fecal microbiota in HISs with CD4^+^ T cell counts <300/mm^3^ was significantly restored (Shannon index: Median 2.557 vs. 2.791, *P* = 0.007; Simpson index: Median 0.168 vs. 0.112, *P* = 0.004) after the introduction of cART, as shown in Fig. 3a, c. On the other hand, among HISs with CD4^**+**^ T cell counts >300/mm^3^, the α-diversity of the gut microbiota decreased slightly after the introduction of cART (Fig. 3b, d), while the variances were insignificant (Shannon index: Median 2.981 vs. 2.934, *P* = 0.179; Simpson index: Median 0.096 vs. 0.119, *P* = 0.082).

Similar to the results described for the α-diversity indices, different alteration patterns in plasma inflammatory biomarkers were observed after cART introduction in the subgroup analysis (Supplementary Table 2). Among HISs with CD4^**+**^ T cell counts <300/mm^3^, the major variances in inflammatory biomarkers after cART primarily manifested as decreased levels of interferon gamma-induced protein (IP)-10 (*P* < 0.001), transforming growth factor (TGF)-α (*P* = 0.019), monocyte chemoattractant protein (MCP)-1 (*P* = 0.002), and IL-4 (*P* = 0.028), despite the increase in IL-5 (*P* = 0.011). For HISs with CD4^**+**^ T cell counts >300/mm^3^, in addition to the decreased IP-10 (*P* = 0.001) and TGF-α (*P* = 0.046) levels consistent with those in the subgroup with lower CD4^**+**^ T cell counts, increased levels of MIP-1β (*P* = 0.001), macrophage-derived chemokine (MDC, *P* = 0.004), fractalkine (*P* = 0.006), epidermal growth factor (EGF, *P* = 0.014), granulocyte colony-stimulating factor (G-CSF, *P* = 0.033), granulocyte-macrophage colony-stimulating factor (GM-CSF, *P* = 0.039), IL-1β (*P* = 0.042), IL-1RA (*P* = 0.049), fibroblast growth factor-2 (FGF-2, *P* = 0.044), and growth-regulated protein (GRO, *P* = 0.046) were observed.

### Gut microbial composition was altered after cART introduction

**Fig. 4:**
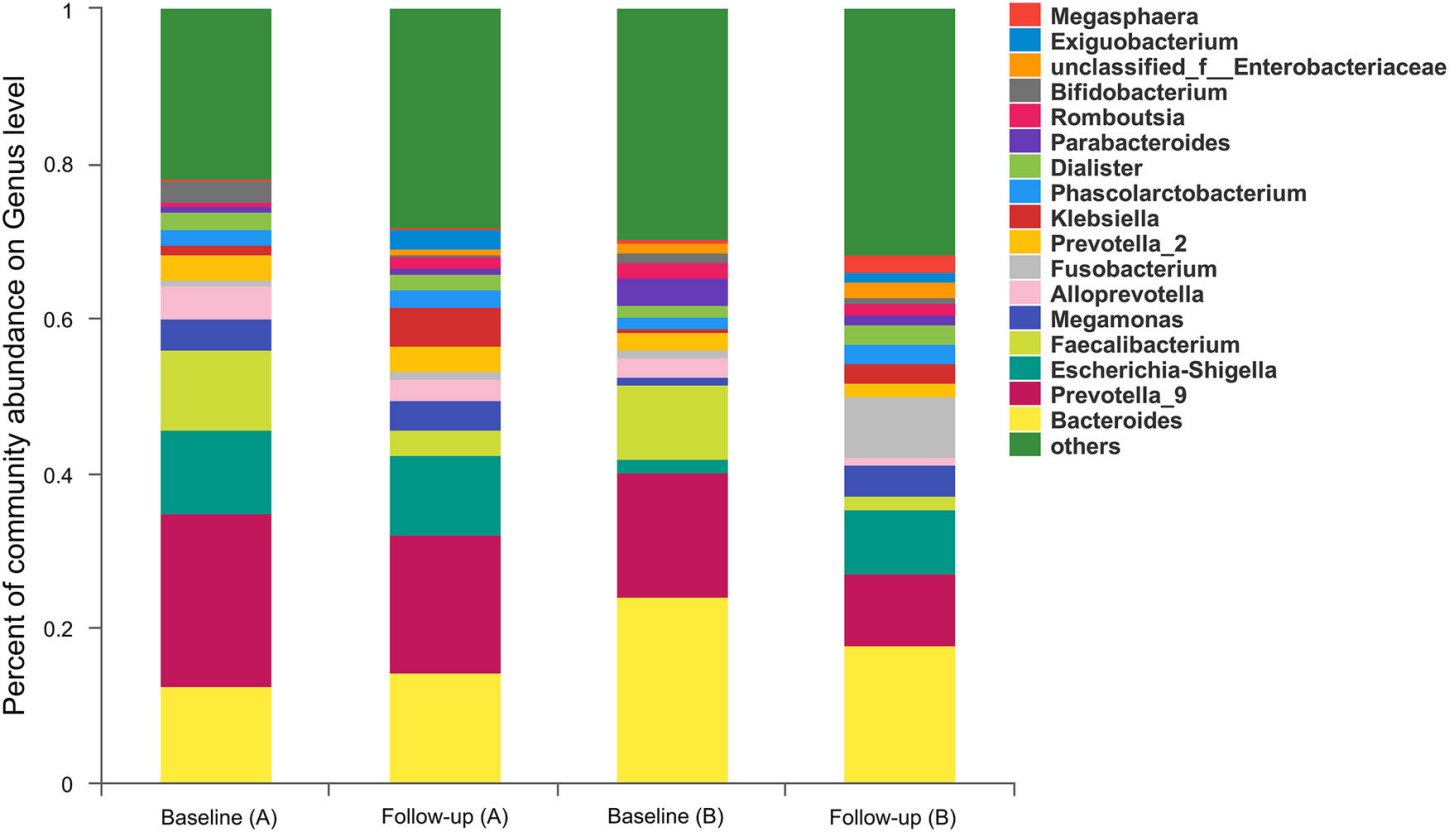
The grouped microbial composition at the genus level at baseline and follow-up. **a** HISs with baseline CD4^+^ T cell counts <300/mm^3^; **b** HISs with baseline CD4^+^ T cell counts >300/mm^3^

With the introduction of cART, the landscape of the gut microbial composition in HISs was distinguishable from that of baseline (Fig. 4). As described for the overall analysis compared with baseline (Table 2), the Proteobacteria (LDA = 4.66, *P* = 0.002) and Fusobacteria (LDA = 4.27, *P* = 0.021) phyla with their sub-taxonomies, including the classes Gammaproteobacteria (LDA = 4.67, *P* = 0.03) and Fusobacteria (LDA = 4.27, *P* = 0.021), the orders Enterobacteriales (LDA = 4.60, *P* = 0.008) and Fusobacteriales (LDA = 4.27, *P* = 0.021), the families Enterobacteriaceae (LDA = 4.60, *P* = 0.008) and Fusobacteriaceae (LDA = 4.27, *P* = 0.020), and the genera *Klebsiella* (LDA = 4.12, *P* < 0.001), and *Fusobacterium* (LDA = 4.27, *P* = 0.017), were relatively increased after cART, while the Bacteroidetes phylum (LDA = 4.78, *P* = 0.027) with its subtaxonomies, including the class Bacteroidia (LDA = 4.78, *P* = 0.027) and the order Bacteroidales (LDA = 4.78, *P* = 0.027), were depleted. Additionally, the relative richness of the Ruminococcaceae family (LDA = 4.63, *P* = 0.006) and the *Faecalibacterium* genus (LDA = 4.55, *P* = 0.001) belonging to the Firmicutes phylum decreased after cART. In the subgroup with baseline CD4^**+**^ T cell counts >300/mm^3^, variations in the gut microbiota composition after cART were similar to those in the bulk analysis, and differences in the order Fusobacteriales, family Fusobacteriaceae and genus *Fusobacterium* became insignificant (Table 2). For HISs with CD4^**+**^ T cell counts  300/mm^3^ at baseline, only relative enrichment of the Bacillales order (LDA = 4.06, *P* = 0.001) with its sub-taxonomies Family_XII_o_Bacillales (LDA = 4.05, *P* = 0.001) and genus *Exiguobacterium* (LDA = 4.05, *P*  = 0.001) were observed after cART administration (Table 2).

**Table 2:** Alterations of gut microbiota composition in HIV positive participants with cART introduction

	Overall (*N*=36)				CD4+ T cell count <300/mm^3^ (*N*=14)				CD4+ T cell count >300/mm^3^ (*N*=22)			
Change	Category	Taxonomy	LDA^a^	*P* ^b^	Category	Taxonomy	LDA^a^	*P* ^b^	Category	Taxonomy	LDA^a^	*P* ^b^
Enrichment	Phylum	Fusobacteria	4.27	0.021	Order	Bacillales	4.06	0.001	Phylum	Fusobacteria	4.52	0.021
	Phylum	Proteobacteria	4.66	0.002	Family	Family_XII_o_Bacillales	4.05	<0.001	Phylum	Proteobacteria	4.77	0.005
	Class	Fusobacteriia	4.27	0.021	Genus	*Exiguobacterium*	4.05	<0.001	Class	Fusobacteriia	4.52	0.021
	Class	Gammaproteobacteria	4.67	0.003					Class	Gammaproteobacteria	4.78	0.004
	Order	Fusobacteriales	4.27	0.021					Order	Enterobacteriales	4.75	0.004
	Order	Enterobacteriales	4.60	0.008					Family	Enterobacteriaceae	4.75	0.004
	Family	Fusobacteriaceae	4.27	0.020					Genus	*Klebsiella*	4.09	0.001
	Family	Enterobacteriaceae	4.60	0.008								
	Genus	*Fusobacterium*	4.27	0.017								
	Genus	*Klebsiella*	4.12	<0.001								
Depletion	Phylum	Bacteroidetes	4.78	0.027					Phylum	Bacteroidetes	4.97	0.010
	Class	Bacteroidia	4.78	0.027					Class	Bacteroidia	4.97	0.010
	Order	Bacteroidales	4.78	0.027					Order	Bacteroidales	4.97	0.010
	Family	Ruminococcaceae	4.63	0.006					Family	Ruminococcaceae	4.73	0.005
	Genus	*Faecalibacterium*	4.55	<0.001					Genus	*Faecalibacterium*	4.59	<0.001

^a^Analyzed by linear discriminant analysis effect size method

^b^Analyzed by paired Wilcoxon’s signed-rank test

## Discussion

Recent studies have described the occurrence of intestinal microbial translocations and microbial dysbiosis in HIV-1-infected individuals^19–23^; these processes are regarded as important pathways for systemic immune activation involving HIV disease progression. The causal relationship between altered intestinal microbiota composition, HIV-1 infection-induced host immune dysfunction and cART remains an open question in HIV research^15^. In this study, we observed that host immune status and cART interactively influenced the alterations of gut microbiota in HISs with viral suppression, which is discussed below.

α-Diversity has been widely measured during evaluations of gut microbial diversity in HISs. In previous studies, compared with non-HIV-1-infected individuals, lower α-diversities of intestinal microbiota were observed in HISs^8,21^, while such discrepancies did not reach significance in other studies^15,17^, and an earlier study reached a contradictory conclusion^23^. In this study, we found that baseline CD4^**+**^ T cell counts were closely associated with α-diversity indices of the intestinal microbiota in HISs. In addition, in subgroup comparisons, significantly lower α-diversity indices were observed in subjects with CD4^**+**^ T cell counts <300/mm^3^. Hence, our findings implied that the conflicting results in previous studies investigating gut microbial diversity in HISs are attributable to the immune-heterogeneous subjects enrolled in different studies.

Currently, findings regarding the role of cART in restoring gut microbiota diversity in HISs are conflicting, as summarized in recent literature reviews^15,24^. In our study, alterations in α-diversity indices after a median of 15 months of cART were not significant in an overall analysis, which corresponded to observations from a previous prospective study^8^. However, in the subgroup with baseline CD4^**+**^ T cell counts <300/mm^3^, the increase in α-diversity indices was significant after cART. Conversely, in subjects with CD4^**+**^ T cell counts >300/mm^3^ at baseline, the α-diversity of the gut microbiota slightly decreased. Because of the established biological link between gut barrier function, microbiome diversity, and systemic inflammation^25–27^, the findings described above were echoed by alterations in specific plasma inflammation biomarkers in this study. In a subgroup of HISs with CD4^**+**^ T cell counts <300/mm^3^, the intensity of systemic inflammation was alleviated after cART, which was indicated by the significant decreases in IP-10, TGF-α, MCP-1, and IL-4. By contrast, in HISs with CD4^**+**^ T cell counts >300/mm^3^, we found several plasma markers, including MIP-1β, MDP, IL-1β, FGF-2, EGF, GRO, G-CSF, and GM-CSF, that were elevated after cART, which closely correlated with host inflammation status^28–34^. In summarizing previous studies, the impact of cART on gut microbiota diversity could be contributed by reconstituting the host’s mucosal immune system as well as direct effects on specific bacterial populations^4,35^. Therefore, in this study, we proposed that intestinal microbial α-diversity in cART-naive subjects with severe immunosuppression was restored via the positive impact of antiviral therapy on immune reconstitution of the host, which was indicated by the alleviation of systemic inflammation, as observed in previous studies^27,36^. In subjects with greater immune competence prior to cART, lower restoration due to immune recovery may be counteracted by the direct influences of cART drugs on gut microbial diversity, which is further discussed below.

HIV-1 infection is associated with host intestinal microbial dysbiosis^6^, while the influence of cART introduction in altering gut microbial composition in HISs has not been fully estimated^19–23^. In our cohort, the alteration patterns in terms of gut microbial composition in HISs with cART introduction were differentially correlated with immune status prior to treatment, which was more drastic in subjects with CD4^+^ T cell counts >300/mm^3^. In light of the findings described above, cART and host immune status appear to interactively contribute to alterations in the gut microbiota composition. The mechanisms by which cART influences the gut microbiota are not fully understood. In a cross-sectional study including 45 HIV-infected patients on three different cART regimens, different cART regimens were associated with diverse profiles in terms of gut microbiota composition, while integrase strand transfer inhibitor-based cART was associated with comparable systemic inflammation and microbial diversity to that of uninfected controls^37^. In a recent study assessing the in vitro antibacterial activity of antiretroviral drugs, efavirenz and zidovudine showed potential inhibition of *Bacillus subtilis* and *Escherichia coli* separately^35^. Given that the regimen containing efavirenz was administered in our study, potential disruption of the intestinal microbiota by efavirenz itself and other cART drugs deserves further consideration and investigation.

We acknowledge certain limitations to this study. First, because of the relatively short follow-up duration, we were unable to observe the long-term influences of cART on the gut microbiome. Additionally, although fecal samples, which were used in our study, are most commonly employed for analysis of the intestinal microbiota, integration analysis based on both fecal and intestinal biopsy samples would be optimal^38^.

## Conclusions

Collectively, the results of our study showed that immune status and cART were the key factors interactively affecting the gut microbiome in HIV-1-infected individuals. All of the above findings provide new insights into interpreting previous studies and the design of future investigations on the intestinal microbiota in HISs.

## Electronic supplementary material

supplement table 1

supplement table 2
