# Supplementary material for: Changes in intestinal microbiota in HIV-1-infected subjects following cART initiation: influence of CD4+ T cell count
Source: Emerg Microbes Infect. 2018 Jun 22;7:113. doi: 10.1038/s41426-018-0117-y (PMC6015051; doi:10.1038/s41426-018-0117-y)
Supplement: Supplementary file 1 — supplement table 1 [file 41426_2018_117_MOESM1_ESM.docx]

| Supplement Table 1. Plasma cytokines measured by MILLIPLEX^®^ kits. |
| --- |
| EGF, FGF-2, Eotaxin, TGF-α, FLT-3L, G-CSF, GM-CSF, IP-10, Fractalkine, IFN-α2, IFN-γ, GRO, MCP-3, MDC, sCD40L, IL-1RA, IL-1α, IL-1β, IL-2, IL-3, IL-4, IL-5, IL-6, IL-7, IL-8, IL-9, IL-10, IL-12 p40, IL-12 p70, IL-13, IL-15, IL-17A, MCP-1, MIP-1α, MIP-1β, TNF-α, TNF-β and VEGF. |
