# Supplementary material for: Changes in intestinal microbiota in HIV-1-infected subjects following cART initiation: influence of CD4+ T cell count
Source: Emerg Microbes Infect. 2018 Jun 22;7:113. doi: 10.1038/s41426-018-0117-y (PMC6015051; doi:10.1038/s41426-018-0117-y)
Supplement: Supplementary file 2 — supplement table 2 [file 41426_2018_117_MOESM2_ESM.docx]

| Supplement Table 2 The alterations of plasma inflammatory markers with cART introduction. | | | | | | | | | | | | | | |
| --- | --- | --- | --- | --- | --- | --- | --- | --- | --- | --- | --- | --- | --- | --- |
| Markers | | Overall | | | | | | CD4+T cell count<300/mm^3^ | | | CD4+T cell count>300/mm^3^ | | |  |
|  | pre-cART^†^ | | | post-cART^†^ | | *P* | | pre-cART^†^ | post-cART^†^ | *P* | pre-Cart^†^ | post-cART^†^ | *P* |  |
| EGF pg/ml | | | 84.7 (16.6-132.3) | | 127.40 (44.83-312.20 ) | | 0.006 | 64.60 (0.83-106.30) | 48.38 (8.39-144.20) | NS | 101.60 (29.24-197.80) | 235.90 (69.94-380.40) | 0.014 |  |
| FGF-2 pg/ml | | | 79.0 (37.02-125.4) | | 97.86 (84.79-141.80) | | 0.015 | 56.26 (0.02-126.60) | 100.30 (80.11-123.90) | NS | 93.03 (50.01-127.50) | 95.40 (85.64-150.10) | 0.044 |  |
| Fractalkine pg/ml | | | 117.2 (65.4-168.0) | | 183.40 (108.20-269.20) | | 0.001 | 76.08 (59.46-174.40) | 197.80 (108.20-284.90) | NS | 129.00 (70.75-148.20) | 183.40 (121.90-245.00) | 0.006 |  |
| G-CSF pg/ml | | | 45.5 (20.6-77.3) | | 82.04 (43.75-109.40) | | 0.019 | 47.18 (19.13-81.53) | 62.21 (27.27-100.40) | NS | 43.75 (23.52-70.75) | 88.47 (60.69-137.40) | 0.033 |  |
| GM-CSF pg/ml | | | 8.2 (2.3-23.1) | | 16.83 (9.33-23.51) | | 0.02 | 2.73 (1.42-17.89) | 13.67 (3.40-19.24) | NS | 11.90 (2.75-23.93) | 17.69 (11.93-33.67) | 0.039 |  |
| GRO pg/ml | | | 1743.00 (741.40- 3342.00) | | 1202.00 (463.70-1742.00) | | 0.022 | 1677.00 (961.70-3254.00) | 1256.00 (656.10-2106.00) | NS | 1809.00 (657.90-3497.00) | 1179.00 (462.20-1600.00) | 0.046 |  |
| IL-11β pg/ml | | | 2.83 (2.19-4.27) | | 3.45 (2.43-5.56) | | NS | 2.63 (1.74-4.49) | 2.20 (1.57-3.80) | NS | 3.33 (2.35-4.06) | 3.92 (2.91-6.04) | 0.042 |  |
| IL-1RA pg/ml | | | 5.37 (0.21-43.92) | | 33.84 (0.12-184.90) | | 0.046 | 0.35 (0.20-21.47) | 0.16 (0.01-50.01) | NS | 16.83 (1.42-151.50) | 102.90 (8.30-276.10) | 0.049 |  |
| IL-2 pg/ml | | | 2.91 (2.23-3.76) | | 2.42 (1.84-3.62) | | 0.046 | 2.87 (2.06-3.94) | 1.94 (1.70-3.37) | NS | 3.24 (2.42-3.76) | 2.48 (1.94-3.93) | NS |  |
| IL-4 pg/ml | | | 32.52 (17.48-51.08) | | 26.60 (12.28-64.62) | | NS | 35.78 (22.25-46.01) | 15.60 (6.61-35.43) | 0.028 | 23.93 (12.74-107.10) | 43.75 (18.15-83.09) | NS |  |
| IL-5 pg/ml | | | 0.99 (0.52-2.30) | | 1.70 (1.02-3.86) | | 0.004 | 0.68 (0.27-1.50) | 0.80 (0.30-3.30) | 0.011 | 1.66 (0.57-4.76) | 2.53 (1.17-7.89) | NS |  |
| IL-7 pg/ml | | | 13.72 (7.26-33.39) | | 23.96 (13.67-37.92) | | 0.046 | 11.56 (4.00-29.34) | 22.75 (8.39-32.26) | NS | 15.20 (8.22-34.46) | 25.16 (18.49-43.21) | NS |  |
| IP-10 pg/ml | | | 1596.00 (858.60-2473.00) | | 453.60 (361.00-842.90) | | <0.001 | 1950.00 (1723.00-3426.00) | 465.20 (327.10-737.20) | 0.001 | 1209.00 (735.80-2223.00) | 447.90 (375.70-911.90) | 0.001 |  |
| MCP-1 pg/ml | | | 405.10 (294.00-655.20) | | 317.00 (231.50-411.50) | | 0.011 | 583.30 (286.80-714.20) | 232.90 (168.10-323.10) | 0.002 | 361.70 (295.90-551.30) | 352.70 (290.40-434.30) | NS |  |
| MDC pg/ml | | | 483.10 (405.20-617.30 ) | | 562.10 (374.50-743.30) | | NS | 489.10 (367.30-621.20) | 395.00 (315.20-520.20) | NS | 477.00 (400.10-645.80) | 661.40 (514.50-895.60) | 0.004 |  |
| MIP-1β pg/ml | | | 28.91 (20.57-40.34) | | 42.40 (27.36-53.34) | | 0.006 | 30.77 (19.87-41.11) | 32.35 (15.70-47.20) | NS | 27.82 (20.57-41.82) | 43.75 (34.52-56.27) | 0.001 |  |
| TGF-α pg/ml | | | 4.05 (2.89-5.71) | | 2.69 (1.41-4.11) | | 0.002 | 4.18 (2.64-5.91) | 1.71 (1.01-2.61) | 0.019 | 3.84 (2.95-5.67) | 3.10 (1.92-4.23) | 0.046 |  |
| TNF-α pg/ml | | | 25.48 (18.14-30.77) | | 17.26 (13.49-23.05) | | 0.001 | 30.00 (23.93-34.04) | 13.67 (7.06-18.14) | 0.002 | 23.93 (17.49-28.90) | 18.61 (14.81-26.71) | NS |  |
| ^†^ Values indicated as median (IQR) | | | | | | | | | | | | | |  |
